# Long noncoding RNAs: new insights in modulating mammalian spermatogenesis

**DOI:** 10.1186/s40104-019-0424-8

**Published:** 2020-02-28

**Authors:** Bahlibi Weldegebriall Sahlu, Shanjiang Zhao, Xiuge Wang, Saqib Umer, Huiying Zou, Jinming Huang, Huabin Zhu

**Affiliations:** 1 Embryo Biotechnology and Reproduction Laboratory, Institute of Animal Science, Chinese Academy of Agricultural Sciences, Beijing, 100193 People’s Republic of China; 2 Tigray Agricultural Research Institute, Mekelle Agricultural Research Center, Mekelle, Ethiopia; 3 Dairy Cattle Research Center, Shandong Academy of Agricultural Sciences, Jinan, 250131 People’s Republic of China

**Keywords:** Germ cell, Long noncoding RNA, Mammalian spermatogenesis, Regulatory pathways, Spermatocytes

## Abstract

Spermatogenesis is a complex differentiating developmental process in which undifferentiated spermatogonial germ cells differentiate into spermatocytes, spermatids, and finally, to mature spermatozoa. This multistage developmental process of spermatogenesis involves the expression of many male germ cell-specific long noncoding RNAs (lncRNAs) and highly regulated and specific gene expression. LncRNAs are a recently discovered large class of noncoding cellular transcripts that are still relatively unexplored. Only a few of them have post-meiotic; however, lncRNAs are involved in many cellular biological processes. The expression of lncRNAs is biologically relevant in the highly dynamic and complex program of spermatogenesis and has become a research focus in recent genome studies. This review considers the important roles and novel regulatory functions whereby lncRNAs modulate mammalian spermatogenesis.

## Introduction

Mammalian spermatogenesis is the process by which male germline cells divide and differentiate into mature spermatozoa [1]. It is an androgen-dependent process that overrides somatic cell and germ cell interactions [2]. Spermatogenesis is a complex physiological process involving the division, differentiation, and meiosis of immature male germ cells into mature haploid spermatozoa in the seminiferous tubules of the testis [3]. This process includes acrosomal formation, elimination of cytoplasm, chromatin reorganization, and flagellum development, replacement of protamines, removal of histone from chromatin material and nuclear formation [2, 3]. Spermatogenesis is also closely related to the Sertoli cells of the seminiferous tubules. Sertoli cells and germ cells produce doublesex and Mab-3 related transcription factor 1 (DMRT1), which is an evolutionary conserved transcriptional factor that regulates tubule morphology and other spermatogonial functions through its Sertoli cell maturation and polarity effects [4]. Lei et al. reported a decreasing trend in the number of cells in seminiferous tubules after silencing lncRNA *H19* (H19 imprinted maternally expressed transcript) [5]. This affected the expression of insulin-like growth factor receptor (IGF-1R) in Sertoli cells and Spermatogenic cells. IGF-1 maintains the survival of several types of stem cells and has essential functions in male reproduction [5]. After the completion of germ cell development into elongated spermatids, the mature spermatids move from the Sertoli cells into the tubule lumen through rete testis until they reach the efferent ducts of the epididymis. The round nucleus of the developing spermatid starts to elongate and condense to achieve a spindle shape during the morphogenesis stage and spermatid develop into long needle-shaped mature sperm [6]. In this complicated developmental process, the mature spermatids execute complex biochemical activities to reach the stage of motile spermatozoa. The dynamic process of spermatogenesis is highly regulated by tissue and cell-specific gene expression. During this precisely regulated biological process, phase-specific gene expression is controlled post-transcriptionally by long noncoding RNAs (lncRNAs) [1, 7]; however, little is known about the regulation of spermatogenesis by lncRNAs in humans [8].

Mammals transcribe a large amount of RNAs; however, only a small fraction (around 2%) of transcripts encode proteins. The remaining large number of transcripts represent small noncoding RNAs and lncRNAs [7, 9]. LncRNAs are a recently discovered class of non-coding transcripts [6], ranging in size from 200 bp to 100,000 bp, and most of them exhibit cell-type-specific expression [10–12]. LncRNAs lack an open reading frame in some or all of their sequence and have no protein-coding ability [7]. They are transcribed in the nucleus and cytoplasm by RNA polymerase II and are spliced, capped, and polyadenylated [13–15].

The molecular functions of lncRNAs have been studied in different cell lines [16, 17] by lncRNA knockdown and overexpression, and include regulation of protein activities, organizational roles, serving as precursors for small RNAs, and modulating transcriptional patterns [18]. LncRNAs are involved in the regulation of many complex cellular and molecular processes during development [6], gene imprinting, and X chromosome inactivation [16]. The majority of lncRNAs are expressed at low levels and generally exhibit low primary sequence conservation. Few studies have demonstrated genetic evidence that mammalian lncRNAs function in in vivo of animal models [6, 19]. LncRNAs often demonstrate restricted time-specific or tissue-specific developmental expression patterns at lower levels than mRNAs. LncRNAs exert their various regulatory functions and biological processes by interacting with splicing factors and recruiting transcription factors. During this process, lncRNAs may alter mRNA splicing and affect gene expression. They may also be involved in transcriptional activation, transcriptional repression, epigenetic modulation, and RNA splicing regulation.

Many lncRNAs have been noted as being involved in spermatogenesis; however, very few of them have been validated and functionally characterized. Thus, the majority of lncRNAs expressed during mammalian spermatogenesis need to be validated and their molecular functions should be specified experimentally. This paper discusses the expression patterns and known functions of certain lncRNAs and their suggested roles in mammalian spermatogenesis. The main purpose of this review was to provide a better understanding of mammalian spermatogenesis in terms of lncRNAs and to explain the classification, expression, and potential roles of lncRNAs in regulating spermatogenesis.

### The developmental processes of mammalian spermatogenesis

The developmental process of mammalian spermatogenesis involves the continuous production of functional sperm, starting from spermatogonial stem cells and involving different cell types. Spermatogenesis involves a series of differentiation of morphologically undifferentiated tissues and cells into highly functional cells, including self-renewing stem cells [2]. The expression of genes and non-coding RNAs during spermatogenesis leads to the production of proteins that maintain the overall housekeeping functions and perform specific processes in the germ cell developmental stages, respectively [20, 21].

There are three principal phases or stages of spermatogenesis: Mitosis, meiosis, and post meiosis stages. Spermatogonial cells undergo mitosis for self-renewal and proliferation, which promotes spermatogonial differentiation. During the second stage, meiosis and genetic recombination occur in the spermatocytes. No further replication occurs and the haploid male germ cells differentiate in the post-replicative stage into spermatids. In mice, the mitotic, meiotic, and post meiotic phases last around 10, 11, and 14 days, respectively. The spermatogenic stem cells differentiate six times during the mitotic phase to form type A spermatogonia, intermediate spermatogonia, and type B spermatogonia. The final division of this phase produces preleptotene spermatocytes that commence meiotic division and undergo the S phase of the spermatogenesis in their last cell cycle. The second meiotic stage is succeeded by two meiotic divisions producing spermatids in the post-meiotic stage. At this stage, DNA replication does not occur and the spermatids develop into fully mature spermatozoa via spermiogenesis by remodeling through the process of acrosome formation, condensation of the nucleus, development of flagellum, and loss of a large portion of the cytoplasm [22–24].

**Fig. 1:**
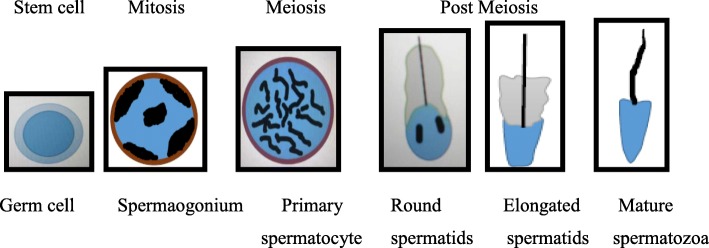
Schematic representation of the developmental processing of spermatogenesis

Thus, spermatogonial stem cells undergo mitotic divisions giving rise to meiotic spermatocytes. Then, the reductive divisions of meiosis lead to the formation of round spermatids (haploid). The round spermatids undergo a series of differentiation to develop into elongated spermatids and then fully develop into mature spermatozoa (Fig. 1).

### Classification of lncRNA

**Fig. 2:**
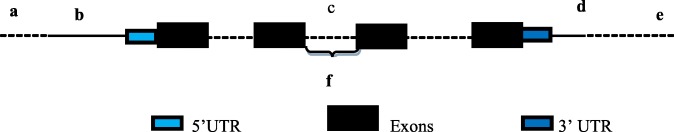
The origin of lncRNA with relative to protein-coding genes structure in different genomic regions. The schematic sketch represents **a** and **e** for Noncoding regions; **b** and **d** for Protein coding genes; **c** for Intronic region and **f** for Cases of intronic retention region

Post-genomic studies have identified a diversity of transcriptional products, such as many small regulatory RNAs and a large number of polyadenylated and non-polyadenylated lncRNAs. These lncRNAs are classified according to their protein-coding loci as intergenic, antisense, intronic, or overlapping lncRNAs. Novel lncRNAs may be similarly classified and subtyped as intergenic, ambiguous, antisense, and intronic lncRNAs according to their genomic locations [22] as shown in Fig. 2. Mattich and Rinn also reviewed the classification of lncRNAs by the relative location of their transcripts according to the adjacent protein-coding genes as intergenic, intronic, bidirectional, overlapping antisense, and overlapping sense lncRNAs [13]. Some lncRNAs are transcribed within long terminal repeats (LTR). Many other lncRNAs do not originate from bidirectional mRNA promoters and may have promoters in their intergenic regions that do not overlap with the LTRs and lncRNA promoters [23].

#### Structural classification of lncRNAs

**Fig. 3:**
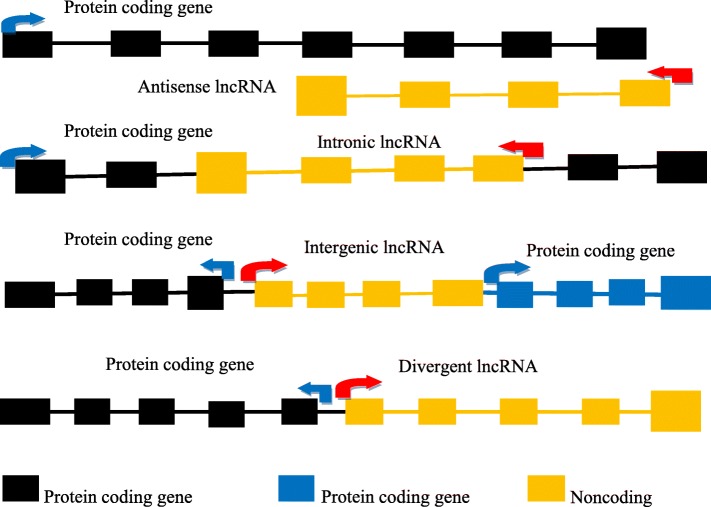
The location of lncRNA in relative to their nearby genes encoding protein

LncRNAs can be categorized into three groups according to their relationship with the adjacent protein-coding genes [24–26] as indicated in Fig. 3.

1) Antisense or sense: These lncRNAs are located and transcribed on the opposite or the same strand of the adjacent protein-coding genes [27]. 2) Convergent (divergent): These lncRNAs have a convergent (divergent) orientation of transcription compared with that of the adjacent protein-coding genes [26]. 3) Intergenic (intronic): These lncRNAs are located between two protein-coding genes, or reside in introns [26, 28].

#### Functional classification of lncRNAs

Most lncRNAs are non-coding, but play diverse roles in many biological processes and are associated with disease occurrence [29]. LncRNAs may function through DNA, RNA, and protein interactions; however, the precise molecular functions of most lncRNAs are unknown. Unlike protein-coding genes, the lncRNA sequence does not have sequence motifs that indicating their function, and their secondary structures are not conserved [25]. This makes it more difficult to predict the function of lncRNAs based on their sequence motifs, conserved sequences, and secondary structures. Current studies indicate that the function of lncRNAs in cell-based studies can through their regulation of, and interactions with, protein-coding genes, microRNAs (miRNAs), and other lncRNAs, using different approaches (Table 1).

**Table 1:** Approaches for lncRNA function prediction

Approaches	Description	References
Comparative genomics	The lncRNA transcripts that are conserved in both human and mouse, and located within or close to a coding gene in < 1 kb distance assumed to have close functional relationship with the neighboring gene. This method of predicting lncRNAs is may not be widely applied to genomic level due to the low conservation potential of lncRNAs.	[30]
Coexpression of lncRNA and coding genes	The lncRNAs and protein coding genes coexpressed in specific biological process regulation. The method is well practiced to predict and identify the enriched function of the lncRNAs at the genomic level.	[11]
Interaction with miRNAs and proteins	The lncRNAs may involve in regulatory network by coordinating with target sites of miRNA. Scientists developed different methods to determine the target sites of miRNA in lncRNAs such as miRcode interface helping the microRNA-lncRNA interaction study across the GENCODE annotated transcriptome, Validated RNA mediated interactions in genome-wide networks in determining the mediatory roles of lncRNA and its interaction and correlation in miRNA, mRNA and proteins which may help in predicting the lncRNA function. This approach is successful for those lncRNAs with known mechanism of interaction between miRNA and protein.	[31]

**Fig. 4:**
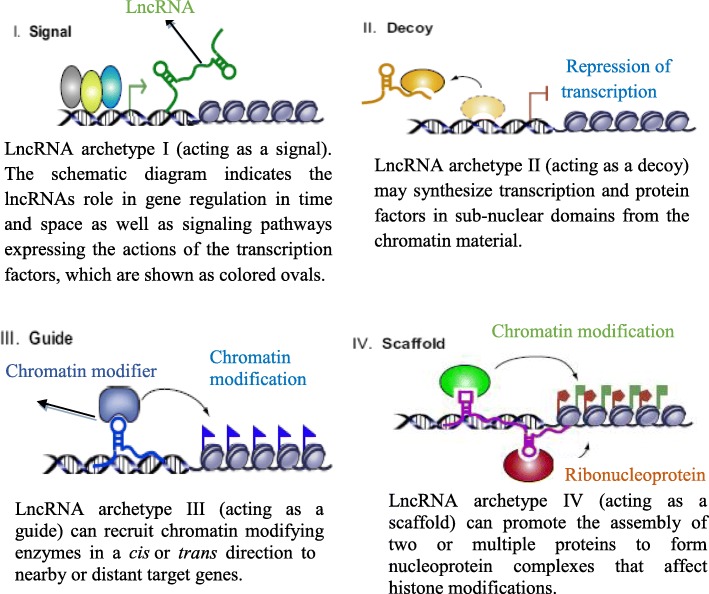
Schematic drawings of the lncRNA archetypes (Adapted from [13, 14])

The lncRNAs use different mechanisms to carry out complex functions and have played some of the following roles [27] as shown in Fig. 4.

I) Signaling: The transcriptional activity of lncRNAs can be described by their response to diverse stimuli, the production of signals, and cell type-specific expression. The molecular mechanism of the majority of the lncRNAs may indicate considerable transcriptional control on a molecular basis by RNA polymerase II. The occurrence of lncRNAs during the developmental processes of specific tissues and cells at a specified time demonstrates the effectiveness of transcriptional control by interpreting the cellular context as well as gene regulation and acts as a molecular signal [14]. The lncRNA-associated signaling pathways indicate the role of transcription factors in gene regulation at a specific time and space for the signaling archetype lncRNAs. De Santa et al. used chromatin immunoprecipitation sequencing (ChIP-seq) to show that lncRNA transcripts can be produced by gene activating enhancers and that their expression correlated positively with the expression level of their neighboring genes, indicating mRNA synthesis regulation [32] as indicated in Fig. 4.
II) Guides: The interaction of lncRNAs with their target molecules may enhance the proper positioning as the transcription machinery on adjacent genes by guiding, in *cis* and in *trans*, for distantly located genes [14]. A lncRNA, as a guide molecule, attaches to the RNA binding complex to guide the complex and regulates the expression of the gene at its genomic locus (see Fig. 4).
III) Decoys: Enhancers and promoters play an important role in transcriptional regulation of lncRNAs, both negatively and positively [33]. Effectors can be negatively regulated by lncRNAs, which function as molecular decoys (see Fig. 4). The telomeric repeat-containing RNA (*TERRA*), which is large noncoding RNA, interacts with the telomerase protein to inhibit its function [34] and the release of the receptor from the DNA of the growth arrest-specific 5 (Gas5) gene via a hairpin sequence motif that represses the glucocorticoid receptor [35], are examples of lncRNAs functioning as a molecular decoys. In addition, the proliferation of spermatogonial stem cells is promoted by lncRNA-AK015322 by acting as decoy for microRNA-19b-3p [36].
IV) Scaffold: The lncRNAs form a distinct complex from two or more proteins to act as a molecular scaffold as shown in Fig. 4. The scaffold complexes may play an important function in controlling and enhancing cellular signals and intermolecular interactions [31]. The scaffold type lncRNAs bind with different domains of the effector components to form a nucleoprotein complex (see Fig. 4). These complexes may be involved in gene activation [28], chromatin modification, and gene repression [37].

### Databases of lncRNAs

Comprehensive datasets play an important role in facilitating the classification, validation, functional prediction, and assessment in low and high throughput lncRNA experiments. Evidence for the biological properties of well-studied and identified lncRNAs has been deposited in specialized databases that have been developed to provide and improve information resources for lncRNA research in the public domain. These developed lncRNA databases have been designed to contain important and comprehensive information about interactions among macromolecules, genomic structure, conservation potential, expression profile, epigenetic modifications, and functional annotations [38].

The lncRNA databases can be categorized as annotation, interaction, and specific databases as shown in Table 2. The annotation databases include NONCODE v4.0, lncRNAdb, LNCipedia, lncRNome, fRNAdb, lncRNAtor, lncRNAMap, and PLncDB. The ChIPBase, NPInter, miRcode, DIANA-LncBase, StarBasev2.0, lncRNA2Target, and lncRNADisease are examples of interaction databases. The specific databases include lnCeDB, NRED, Linc2go, and lncRNASNP [49–59].

**Table 2:** Descriptions of widely used lncRNA databases

Tools	Web address	Description
EVlncRNA, [39]	http://biophy.dzu.edu.cn/EVLncRNAs	It provides 77 species of functionally annotated long noncoding RNAs validated for disease specific roles through low put experiments and deals with evolution and disease association of lncRNAs.
LNCipedia [40]	https://lncipedia.org/	It is one of the lncRNA reference gene database containing a total of 127802 unique transcripts, 56946 unique genes, 2482 lncRNA articles and 1555 lncRNA annotated genes including their functional information.
NONCODE [41]	www.noncode.org/	It is one of the comprehensively compiled databases which is integrated database with complete collection and annotation of lncRNAs but doesn’t include tRNAs and rRNAs. This database contains 17 species and 73,372 lncRNAs as well as literatures and public data bases.
LncRNADisease [42]	http://cmbi.bjmu.edu.cn /lncrnadisease	It provides a systematic collection of lncRNA and circular RNA disease association, transcriptional regulation of lncRNAs, miRNAs and mRNAs. It is one of the important database used in the clinical application for lncRNAs related studies.
LncRBase [43]	http://bicresources.jcBose.ac.in/zhumur/	It is enriched resource of lncRNA transcripts of human (133,361 entries) and mouse (83,201 entries) of 14 distinct subtypes with 8507of mouse and 14,813 of human newly annotated noncoding RNAs, piwi interacting RNAs and microRNAs including their regulation and association with many other genomic elements.
LncRNAdb [44]	www.lncrnadb.org/	It is a database with comprehensive collection of lncRNAs with their information of biological functions in eukaryotes and regulatory role of messenger RNA. It contains most of the relevant information about the RNA, annotation, target tissues of expression and associated diseases.
LncCeRBase [45]	http://lnccerbase.it1004.com	This database is developed for the competing endogenous RNAs (ceRNAs) encompassing 432 interactions of lncRNA-miRNA-mRNA with 130, 214 and 245 of lncRNA, miRNA, and genes respectively. It also have interactions of lncRNA-miRNA-mRNA associated with signaling pathways to explore the lncRNA regulatory mechanisms. It is designed specifically to studied and validated human ceRNAs.
RNAcentral [46]	http://rnacentral.org	It is an integrated database that compiled sequences of noncoding RNA of all RNA types and organisms serving a single entry for all RNA sequence searches. This comprehensive database has 22 collaborating databases from specialized non-coding RNA resources.
LncRNome [47]	http://genome.igib. res.in/lncRNome	Provides an outlook on annotation of more than 18000 transcripts of the different classes of lncRNAs including the intergenic lncRNAs, antisense lncRNAs, intronic lncRNAs, overlapping lncRNAs and processed pseudogenes. It is designed to provide relevant information for human lncRNAs and their respective function. This database focuses on compiled information about sequence, structure, genomic loci, motifs, expression and associated diseases of lncRNAs.
NRED [48]	http://jsm-research.imb.uq.edu.au/NRED	It provides relevant information on gene expression of lncRNA in mammals especially in human and mouse. This database consists of microarray data and in situ hybridization data and serves as an important resource to the scientific community to study and understand the lncRNAs.

### Identification of lncRNAs during spermatogenesis

LncRNAs can be identified and functionally characterized systematically in different ways [6]. Genome-wide transcriptome analyses [60, 61] RNA-Seq approach [62], RNA capture sequencing [63], and the development of efficient gene knock out technologies like the Clustered regularly interspaced short palindromic/Cas9: CRISPR-associated protein-9 nuclease (CRISPR/Cas9) with a homologous recombination system [6] could expand our understanding of lncRNAs’ biological functions in spermatogenesis. The optimized CRISPR system plays an important role in targeting gene replacement and knock out up to 92 kb to study gene function and is successful because of its low rate of off-target effects*.* Phylogenetic, conservational, and comparative genomic analyses will also help to determine the evolution and origin of testis enriched lncRNAs. Cell-based in vitro studies of genomic loss of function [64], including targeted in vivo lncRNA silencing and deletion revealed the mechanisms of lncRNAs in cellular processes and development [6, 65, 66]. See the descriptions of the other identification methods of lncRNA in Table 3.

**Table 3:** Descriptions of other methods to identify lncRNAs

Type	Methods [Ref]	Description
Experimental methods of lncRNA identification	Microarray [67]	It uses Computational and annotation pipeline to determine the expression and regulation potential of lncRNA transcripts. It has higher efficiency in high put lncRNA analysis. However, its detection potential is low due to its low sensitivity and low expression level of the lncRNA
	SAGE [60, 68]	The serial analysis of gene expression (SAGE) is a technology which identifies the lncRNA known and unknown transcripts by producing short sequence tags and is the highly effective method to study lncRNAs but, it is expensive and not applicable in large scale researches.
	EST [69, 70]	Expressed sequence tag (EST) is a cDNAs short subsequence generated from cDNA clone by one shot sequencing to discover novel and functional transcripts of lncRNA in mammalian. This public database helps to search the transcripts in the intergenic regions of genes and reconstruct lncRNA transcript assemblies.
	RNA-Seq [71]	It is a shotgun sequencing of whole transcriptome in the next generation sequencing technologies and is used to identify novel lncRNA transcripts and gene expression analysis.
	RNA-IP [72]	RNA-immunoprecipitation one of the latest techniques that used antibodies of protein to discover and isolate the lncRNA that interacts with protein complexes or specific proteins by constructing cDNA library and deep sequencing of lncRNAs.
	Chromatin Signature Based Approach [11]	Is a method that do not target directly on the RNA transcripts but directly involves in the identification of lncRNA expression regulation mechanisms using Chromatin signatures and their regulation factors.
Computational methods of lncRNA identification	ORF Length Strategy [73]	This strategy is a method used to differentiate the lncRNA from the mRNA by the Open Reading Frame (ORF) length cutoff based on codons length.
	Sequence and Secondary Structure Conservation Strategy [74–76]	This strategy is used to differentiate the non-coding genes from the protein coding genes by using different methods and strategies such as conservation potential, measure of coding potential, codon substitution frequency scores, reading frame conservation and PhyloCSF. The other methods that are used to explore the RNA secondary structure conservation include the programs of QRNA, EvoFOLD and RNAz.
	Machine Learning Strategies [77]	Due to the complexity of lncRNAs, a new machine learning systems have been increasingly developed such as SVM (support vector machine) based machine learning technique like CONC (coding or non-coding), and other models to integrate and utilize various protein features to distinguish the lncRNAs from mRNAs.

### The expression of lncRNAs during mammalian spermatogenesis

Mammalian spermatogenesis plays an important role in fertility and the continual replacement of species from generation to generation. Previously, many studies have analyzed the molecular mechanisms of mammalian spermatogenesis, focusing on microRNAs, Piwi-interacting RNAs (piRNAs), and protein-coding genes; however, limited studies have been performed on lncRNAs in mammalian spermatogenesis. Some studies reported testis and germ cells expression profiles of lncRNAs in different ages and stages of development [52]. Bao et al. analyzed lncRNAs in fetal and postnatal mouse testes using microarray-based profiling to investigate the novel links between lncRNAs and their targets [52]. The results suggested the involvement of lncRNAs in gene regulation at transcriptional and post-transcriptional levels. Sun et al. conducted a microarray analysis to assess the lncRNA expression profiles during postnatal development in mouse testis [21]. Song et al. explored the testis-specific lncRNAs and their expression in adult mouse testis using experimental and computational methods [53] and reported highly expressed lncRNAs in adult testis. Wichman et al. analyzed the dynamic expression pattern of lncRNAs in the testis that escapes the meiotic sex chromosome inactivation (MSCI) [54]. The X- and Y- linked lncRNAs showed higher expression in pachytene spermatocytes, suggesting their involvement in escape from MSCI. In that study, RNA-Seq analysis confirmed that a small number of mRNAs showed higher expression patterns in the pachytene stage, indicating that few mRNAs escape MSCI.

Recent studies have assessed the lncRNA expression during spermatogenesis. Many lncRNAs show different expression levels and tissue specificities in different stages of mammalian spermatogenesis [55], suggesting that they have various functions in different biological processes, such as in the regulation of gene silencing, cell division, gonadogenesis, and sex determination. Zhang et al. reported the expression of lncRNAs in human normal sperm and in asthenozoospermic patients. The results suggested that enriched lncRNAs have an important role in sperm motility [56]. Some of the lncRNAs, such as *lnc32058*, *lnc09522*, and *lnc98487* are upregulated in asthenozoospermic sperm, exhibiting specific expression patterns in sperm and testis and are related to the progressive motility of sperm, providing insights into the causes of male infertility [56]. Zhang et al. revealed that the low expression of lncRNA *HOTAIR* (Hox transcript antisense intergenic RNA) in the spermatozoa of patients with asthenozoospermia and oligoasthenozoospermia, which might be related with the motility and vitality of sperm [57]. Evolutionary conservation analysis of hsa-lncRNA12238 demonstrated its highest expression in the human testis, indicating its involvement in spermatogenesis, sperm-egg recognition, and other reproductive processes [58].

Liang et al. analyzed the overall sequential expression of lncRNAs in four specific stages of spermatogenesis, mainly in spermatogonial stem cells (SSC), type A spermatogonia (AS), pachytene spermatocytes (PS), and round spermatids (RS) in mice using microarray analysis. They reported that intergenic lncRNAs were highly expressed in the germ cell types, acting as tissue-specific lncRNA genes and as housekeeping genes, sense overlap lncRNA, antisense lncRNA, and bidirectional lncRNA [59]. Furthermore, correlation analysis between mRNAs and lncRNAs showed high correlation coefficients and suggested coordinated changes in their expression during the biogenesis and function of male spermatogenesis. A similar investigation reported the expression of antisense lncRNA *1700108J01Rik* and long intergenic noncoding RNA *1700101O22Rik* [53] in meiotic prophase and round spermatid stages in testicular germ cells. Similarly, Dai et al. reported higher expression of lncRNA-testicular cell adhesion molecule 1 (lncRNA-*Tcam1*) in round spermatids than in somatic cells or germ cells in mice, with lncRNA-*Tcam1* being localized in 45% of the cells found in testis [55]. See the expressions of some lncRNAs in Table 4.

**Table 4:** The expression of certain long noncoding RNAs during the progression of mammalian spermatogenesis

LncRNA name	Location^a^/Length	Expression level [Ref]
Gm11837	Chr.4: 14929908–14953030 (23122 bp)	Highly expressed in adult mouse testis [53]
LncRNA-Gm2044	Chr.7: 139957005–139958039 (1034 bp)	Highly expressed in spermatocytes [78]
LncRNA H19	Chr.7:142575529–142578143 (2.6 kb)	Highly expressed in testis [5]
Meiotic recombination hot spot1 locus (Mrhl)	Chr.8:85994245–85996642 (∼2.4 kb)	Expressed in spermatogonial Gc1-Spg (derived from type B spermatogonia) cells [79]
AK007004	Chr.12:82932520–82939155	Low expressions in male germ cells [80]
AK015322	Chr.12:26814371–26834873	Highly expressed in spermatogonial stem cells [36]
*1700101O22Rik*	Chr.12: 7372039–7380330 (8291 bp)	Highly expressed in testicular germ cells in the meiotic prophase and round spermatocyte stage of spermatogenesis [53]
lncRNA4667	Chr.13:23396074–23397418 (1.3 kb)	Highly expressed in round spermatids [55]
*1700108J01Rik*	Chr.14:122229905–122233638 (3733 bp)	Specifically expressed in testis and highly expressed in testicular germ cells during testicular spermatogenesis [53]
LncRNA HOTAIR	Chr.15:102944062–102947730 (∼3.7 kb)	Low processes in the spermatozoa of patients with asthenozoospermia and oligoasthenozoospermia [57]
Metastasis-associated lung adenocarcinoma transcript 1 (*Malat1*)	Chr.19:5795690–5802671 (∼7 kb)	Expressed in sperm cells and intestinal cells of testis [19]
LncRNA-Tsx	Chr.X	Expressed in meiotic germ cells [81]
LncRNA-Xist (X-inactive specific transcripts)	Chr.X:103460373–103483233	Highly expressed in male germ cells [81]
*Tslrn1 (1700019B21Rik)*	Chr.X:62510539–62527011 (∼16.5 kb)	Highly expressed in pachytene spermatocytes [54]
*Tesra*	4435 bp	Highly expressed in the nuclei of pachytene spermatocytes [82]
LncRNA*-Tcam1*	2.4 kb	Expressed in mouse male germ cells [55]
LncRNA033862	∼6.4 kb	Highly expressed in mouse spermat- ogonial stem cells especially in early spermatogonia [80]

^a^The chromosomal location is in the mouse genome

Therefore, increasing amounts of research have demonstrated the dynamic changes in gene expression in the complex process of spermatogenesis. The findings of these studies provide new insights into lncRNAs expression levels and their tissue specificity in testicular spermatogenesis, suggesting their specific roles in mammalian spermatogenesis.

### Functional roles of lncRNAs during mammalian spermatogenesis

LncRNAs are mRNA-like transcripts of greater than 200 nucleotide sequences exhibiting little or no protein-coding ability [83] and represent poorly understood ncRNAs. However, lncRNAs and protein-coding genes exhibit conserved co-expression networks during the division and differentiation of male germline stem cells into mature sperm [84]. The testis-specific lncRNAs evolved faster and showed higher conservation of post-transcriptional sequences during germ cell differentiation. The lncRNAs regulated genes are mostly associated with metabolic and reproductive activities, indicating the influence of lncRNAs on the control of gene expression during germ cell development. The transcriptional regulatory functions of testis-specific lncRNAs were predicted to control the expression of genes in proximal and distal positions [6].

Wen et al. reported the partial and full rescue of testis-specific lncRNAs in a trans-configuration using transgenes, indicating their DNA regulatory functions *in trans* during late spermatogenesis [6]. This study also indicated the functional role of certain testis-specific lncRNAs in the control of nuclear condensation efficiency, shaping, and spermatid differentiation or individualization. Other lncRNAs are via an *in cis*-mediated mechanism to regulate neighboring gene expression [85, 86]. Song et al. reported the cytoplasmic distribution of testis-specific lncRNAs involved in post-transcriptional gene regulation [53].

Mammals exhibit condensation and remodeling of their chromatin material during late spermatogenesis by omitting excess cytoplasm and replacing histones with protamine for spermatid individualization, leading to a highly compact sperm nucleus [64]. Enhancer-associated lncRNAs participate in transcriptional activation by acting over long distances on distal promoters associated with protein factors and the modulation of chromatin structures [87]. Song et al. revealed the cytoplasmic distribution of *1700108J01Rik* and *1700101O22Rik* using in situ hybridization, suggesting their function in post-transcriptional gene regulation mainly in the nucleus; however, they are not involved in epigenetic and transcriptional regulation [53]. Similarly, Zhang et al. reported the post-transcriptional role of lncRNA *Dmrt1* on chromosome 5 in spermatogenesis and testis development in mice [65]. A study by Liang et al. revealed that the identified specific lncRNAs in spermatogonial stem cells play important roles in maintaining these cells, and some of them are involved in the regulation of differentiation of specific stages of testicular spermatogenesis and germ cell meiosis [80]. The authors concluded that the transcription mechanism of the mRNA and lncRNA play a key role in the differentiation and meiosis of mouse male germ cells. This study also provided comprehensive data on the coordinated changes in total lncRNA/mRNA transcription regulation, suggesting their importance in reproductive disorder diagnosis and treatment. The role of some lncRNAs in mammalian spermatogenesis is shown in Table 5.

**Table 5:** List of certain long non-coding RNAs, and their role in mammalian spermatogenesis

LncRNA name	Length	Chromosomal location*	Function [Ref]
HongrES2	1.588 kb	Chr. 5	Responsible for normal sperm capacitation in the epididymis [88]
LncRNA-Dmrt1	–	Chr.5	Possibly involved in the switching between mitosis and meiosis of developing germ cell [88]
LncRNA H19	2.6 kb	Chr.7	Affects the expression of IGF-1R by regulating the IGF-1 pathway [5]
LncRNA Gm2044	1034 bp	Chr. 7	Inhibits Utf1 mRNA translation and plays a potential role in spermatogenesis [78]
Meiotic recombination hot spot1 locus (Mrhl)	∼2.4 kb	Chr. 8	Inhibits the Wnt signaling pathway during spermatogenesis by interacting with p68 in spermatogonia [89, 90]
*Tesra*	4435 bp	Chr.9	Activation of *Prss42/Tessp-2* gene [82]
LncRNA-HSVIII	–	Chr.9	Participate in the activation of the *Prss42/Tessp-2* gene promoter [82]
LncRNA033862	6384 bp	–	Regulates *Gfra1* expression levels and spermatogonial stem cells fate [17]
LncRNA-Tcam1	2.4 kb	Chr.11	Important for the immune response during spermatogenesis [55]
Malat1	∼7 kb	Chr.11	Plays a potential *cis*-regulatory role of transcription [19]
AK015322	∼20.5 kb	Chr.12	Promotes the proliferation of spermatogonial stem cells [36]
LncRNA4667	1.3 kb	Chr.13	A marker for round spermatids identification in mice [55]
LncRNA HOTAIR	∼3.7 kb	Chr.15	Regulates HoxD genes expression in *trans by* interacting with chromatin modification complexes [28]
LncRNA Neat1	3.2 kb	Chr.19	Plays an important role in the maintenance and assembly of nuclear speckles of mammalian cells [90]
LncRNA Jpx	–	Chr.X	Regulates the X-chromosome inactivation (Xi) switch [91]
LncRNA Tsix	–	Chr.X	Regulates the X-chromosome activation (Xa) switch [91]
LncRNA Tsx	–	Chr.X	Involved in apoptosis in meiotic division during spermatogenesis [81]
LncRNA-Xist	⁓17 kb	Chr.X	Involved in X chromosome inactivation [81].
LncRNA NLC1- C	–	–	Has a potential role in male fertility and regulates miRNA expression [78]
LncRNA Spga	–	–	Involved in spermatogonial differentiation *in vitro* [60]

* The chromosomal location is in the mouse genome

### Molecular signaling pathways enriched in lncRNA modulated spermatogenesis

LncRNAs play an important role in diverse biological processes by interacting with regulatory pathways of epigenetics [13]. Zhang et al. revealed that the differentially expressed lncRNAs between normal sperm and sperm from asthenozoospermic patients showed involvement of lncRNAs in different processes of spermatogenesis, as determined using gene ontology and pathway analysis [56]. The lncRNA *Mrhl* (Meiotic recombination hot spot1 locus) interacts with the p68 (DEAD-box helicase 5) protein and binds to the *SOX8* (SRY-box 8) promoter to inhibit the Wnt signaling pathway in mouse spermatogonial cells to ensure normal sperm production [66]. The apoptosis and proliferation of male germ stem cells are regulated by lncRNA *H19* through the IGF-1 signaling pathway [5]. The suppressing effect of lncRNA *Gm2044* is mediated by the miR-202-Rbfox2 molecular signaling pathway in the proliferation of human testicular embryonic carcinoma cells. This provides new clues to understand male reproduction and the importance of lncRNAs in the miR-202-Rbfox2 molecular signaling pathway in the pathogenesis and treatment of male infertility [92]. Wichman et al. postulated the influence of some lncRNAs in fertility and spermatogenesis and explored the dysregulation of specific lncRNAs, which is a novel mechanism indicating fertility and low sperm quality, helping to identify new therapeutic strategies and biomarkers [54].

## Conclusions and future directions

Advances in transcriptomic studies, especially in RNA sequencing, have increased the identification of lncRNAs. Recently developed computational methods for lncRNA identification and functional prediction have increased our knowledge of the various functions of lncRNAs. This has helped us to understand the complex lncRNA associated processes in various systems, lncRNAs’ molecular functions, and their mechanisms. LncRNAs have an important function in mammalian spermatogenesis; however, the specific roles of only a few lncRNAs have been determined. Therefore, it remains challenging to develop accurate and efficient methods to characterize lncRNAs and lacks detailed information about the specific roles of lncRNAs and the interaction between lncRNAs, genes, proteins, and other molecules in specific tissues and cells. Therefore, developing more powerful computational methods and comprehensive databases, and performing further experimental studies are required to determine the molecular functions, mechanisms, specific expression, and tissue specificity of lncRNAs in different cellular developmental processes. Future studies combining different approaches in lncRNA research are likely to provide a comprehensive understanding and exciting insights into this rapidly evolving field of study in complex biological processes.

The localization of lncRNAs in the vicinity of protein-coding genes and the existence of conserved lncRNAs indicate closer interactions and interrelated functions of lncRNAs in the mammalian genome. The conserved lncRNAs might also represent new prognostic biomarkers and novel diagnostic options to design therapeutic drugs. Recent advances in nucleic acid drug development have also started to provide successful solutions to male sterility problems, male contraceptives, and will allow the exploration of lncRNAs viable targets to combat male infertility, pathogenesis [92], and other pathologies.

Currently, rapid advances are being made in lncRNA pathway studies and understanding their molecular functions [5, 92, 93]. Researchers have developed alternative methods to silence the genes; however, they have failed to develop effective methods to express therapeutic genes, which remains challenging. LncRNAs could provide the best option to target a defined subset of genes found within their vicinity, in either proximal or distal positions. Although such advances in therapeutic technologies are encouraging, there are several challenges that must be addressed before their clinical applications. LncRNAs are considered to be suitable for therapeutic targets because of their presence in specifically defined developmental stages and cell-specific expressions. Therefore, further research should focus on the overall expression, off-target effects, the status of the immune response, and other issues related to lncRNAs.

## Data Availability

Not applicable.

## Abbreviations

AS: Type A spermatogonia
Cas9: CRISPR-associated protein-9 nuclease
cDNA: Complementary DNA
ceRNAs: Competing endogenous RNAs
CHIP-seq: Chromatin immunoprecipitation sequencing
CONC: Coding, noncoding
CRISPR: Clustered regularly interspaced short palindromic
DMRT1: Doublesex and mab-3 related transcription factor 1
DNA: Deoxyribonucleic acid
EST: Expressed sequence tag
Gas5: Growth arrest-specific 5
HOTAIR: Hox transcript antisense intergenic RNA
IGF: Insulin-like growth factor
ISH: In situ hybridization
kb: Kilo base
LncRNA: Long noncoding ribonucleic acid
LncRNA-*Tcam1*: LncRNA-testicular cell adhesion molecule 1
LTR: Long terminal repeat
*Malat1*: Metastasis-associated lung adenocarcinoma transcript 1
miRNA: microRNA
*Mrh1*: Meiotic recombination hot spot1 locus
mRNA: messenger RNA
MSCI: Meiotic sex chromosome inactivation
*Neat1*: Nuclear enriched abundant transcript
ORF: Open reading frame
RNA: Ribonucleic acid
rRNA: Ribosomal RNA
RS: Round spermatids
SAGE: Serial analysis of gene expression
SP: Pachytene spermatocytes
SSC: Spermatogonial stem cells
SVM: Support vector machine
*TERRA*: Telomeric repeat-containing RNA
tRNA: Transfer RNA
*Tslrn1*: Testis specific lncRNA1
Tsx: Testis-specific X-linked
UTR: Untranslated region
Xist: X-inactive specific transcripts
